# Dr. Orrin I. Hall

**Published:** 1908-08

**Authors:** 


### OBITUARY

---

Dr. Orrin I. Hall, of Zumbrota, Minn., died at his home in that city June 25, 1908, aged 66 years. He graduated in medicine at the University of Buffalo in 1873.

